# Deep Learning Reconstruction of Diffusion-weighted MRI Enables Shorter Examination Times While Maintaining Image Quality in Head and Neck Imaging

**DOI:** 10.1007/s00062-025-01604-6

**Published:** 2026-01-07

**Authors:** Haidara Almansour, Jan Michael Brendel, Christoph Neatu, Sebastian Gassenmaier, Judith Herrmann, Sebastian Werner, Vitali Koch, Omar Darwich, Elisabeth Weiland, Thomas Benkert, Sebastian Altmann, Andrea Kronfeld, Ahmed E. Othman, Konstantin Nikolaou, Saif Afat

**Affiliations:** 1 Department of Neuroradiology, University Medical Center of the Johannes Gutenberg University Mainz, Mainz, Germany; 2 Department of Diagnostic and Interventional Radiology, Tuebingen University Hospital, Tübingen, Germany; 3 Cluster of Excellence iFIT (EXC 2180) “Image Guided and Functionally Instructed Tumor Therapies“, University of Tübingen, Tübingen, Germany; 4 Department of Diagnostic and Interventional Radiology, University Hospital Frankfurt, Frankfurt am Main, Germany; 5 MR Applications Predevelopment, Siemens Healthineers AG, Forchheim, Germany

**Keywords:** Diffusion-weighted imaging, MRI, Deep Learning Reconstruction, Head and neck, Staging, acceleration

## Abstract

**Rationale and Objectives:**

Diffusion-weighted imaging (DWI) of the head and neck is essential for various clinical applications but is often hampered by artifacts and reduced image quality. Deep learning (DL) reconstruction has the potential to enhance the quality of head and neck DWI. This study aims to evaluate the performance of an accelerated, DL-reconstructed DWI (DWI_DL_) in terms of image quality and diagnostic confidence.

**Materials and Methods:**

This retrospective study included patients who underwent clinically indicated head and neck DWI at 1.5 T and 3 T between August 2023 and January 2024 at a tertiary care center. Imaging was performed at low b‑values (0 or 50 sec/mm^2^) and high b‑values (800 sec/mm^2^), and apparent diffusion coefficient (ADC) maps were computed. After acquiring standard single-shot echoplanar imaging DWI sequences, the raw MR datasets underwent simulated acceleration by reducing the number of signal averages. These accelerated exams were then reconstructed using a novel DL-based algorithm that combined DL-based k‑space to image reconstruction with DL-based super-resolution processing (DWI_DL_). Three readers analyzed the images using a visual Likert score to evaluate image sharpness, artifacts, noise, overall image quality, and diagnostic confidence. Comparisons were made using the Wilcoxon signed-rank test. A quantitative analysis of signal-to-noise ratio (SNR), contrast-to-noise ratio (CNR) and apparent diffusion coefficient values (ADC) was also performed.

**Results:**

The study included 30 patients (mean age, 55 ± 19 years; range, 24–84; 18 men) with various pathologies. Scan times were reduced by 67% at 1.5 T and up to 55% at 3 T. The quantitative analysis revealed a minimal but statistically significant decrease in SNR and CNR in the deep learning-reconstructed images (*p* = 0.002 and *p* < 0.001, respectively). However, readers reported no significant differences between DWI and DWI_DL_ regarding image quality parameters or diagnostic confidence for both low and high b‑value images, as well as the ADC (all *p* > 0.05).

**Conclusion:**

DL reconstruction of head and neck DWI is feasible, significantly reducing examination time without compromising image quality or diagnostic confidence. This technique enables accelerated and effective diagnostic DWI of the head and neck.

## Introduction

Diffusion-weighted imaging (DWI) is an instrumental part of magnetic resonance imaging (MRI) of the head and neck, which could be considered indispensable in a wide variety of clinical applications. This technique is routinely used in the detection and characterization of tumors, staging of lymph nodes, assessment of treatment response and early detection of recurrence after treatment [1]. The fundamental principle of DWI is the depiction of Brownian motion of water protons in tissues [1, 2]. This motion is quantified by the apparent diffusion coefficient (ADC), which is contingent upon the selected b‑value. Low b‑values usually depict microperfusion and minimal diffusion. Thus, high b‑values are essential to mirror true diffusion in biological tissues [1]. However, high b‑values are accompanied by a low signal-to-noise ratio (SNR), which needs to be compensated by acquiring multiple signal averages and longer examination time [3].

Clinically, echo planar imaging (EPI) is considered one of the most versatile and useful sequences for DWI due to its robust nature against phase errors induced by motion, a relatively shorter acquisition time and relatively high signal [4–6]. However, single-shot EPI-DWI is sensitive to static field inhomogeneities, which could compromise signal, fat saturation, geometric distortion, and eventually diagnostic image quality and reduced diagnostic confidence [4–8], especially in areas with complex anatomical geometry such as the head and neck.

Given the rising demand for MRI examinations within an aging demographic, coupled with a limited acceptance of prolonged scanning sessions, it is imperative to streamline the MRI process without sacrificing diagnostic image quality [9]. Fast MRI examinations, conforming to the highest quality standards, are indispensable for maintaining an efficient MR workflow. In this context, one of the major drawbacks of DWI is the time-consuming image acquisition due to repetitive measurements. To overcome distortion problems, read-out segmented EPI sequences are an alternative [4, 10], which leads to even prolonged acquisition time [4, 10].

In this context, multiple techniques were developed to accelerate DWI. Parallel imaging techniques are commonly used, but they are limited by a reduced signal-to-noise ratio and increased image noise [8, 11, 12]. Therefore, parallel imaging techniques are confined to low acceleration factors [9, 11]. Recently, deep learning reconstruction has been suggested as the next frontier in improving the image quality of DWI in abdominal imaging [9, 13], prostate imaging [3, 14] and neuroimaging [15]. In head and neck imaging, a retrospective study examined the impact of DL reconstruction on image quality, but without shortening examination time [8].

The purpose of this study was to investigate the performance and clinical feasibility of accelerated and deep learning reconstructed diffusion-weighted imaging (DWI_DL_) of the head and neck.

## Materials and Methods

### Study Design

The institutional review board approved this retrospective, single-center study with a waiver of informed consent. All study procedures were conducted in accordance with the ethical standards as laid down in the 1964 Declaration of Helsinki and its later amendments.

**Fig. 1:**
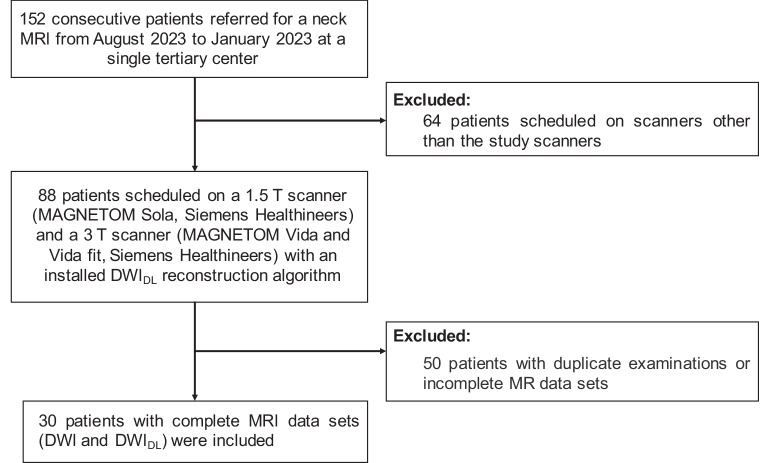
Flowchart detailing the inclusion and exclusion of the study’s cohort. *DWI* Diffusion-weighted imaging. *DL* deep learning. *DWI*_*DL*_ deep learning-reconstructed diffusion-weighted imaging

Adult patients who underwent a clinically indicated MRI of the head and neck region on a 1.5 T scanner (MAGNETOM Sola; Siemens Healthineers, Erlangen, Germany) or a 3 T scanner (MAGNETOM Vida and Vida Fit; Siemens Healthineers, Erlangen, Germany) and whose raw data sets were reconstructed with the DWI_DL_ reconstruction algorithm between August 2023 to January 2024 were included in this study. Figure 1 shows the inclusion process of the study sample. Table 1 shows the demographics of the included patients and indications of the MRI examination. The research implementation of DWI_DL_ was provided by Siemens Healthineers. The authors, who are not affiliated with Siemens Healthineers, had full control of patient data.

**Table 1:** Patient characteristics

Characteristic	Value (*n* = 30)
**Demographics**	
Age [years]*	55 ± 19
Age range [years]†	24–84
Women	12/30 (40)
Men	18/30 (60)
**Indication for the MRI Examination**	
*Evaluation of an indeterminate cervical mass*	5/30 (17)
*Cervical lymphadenopathy without a clear cause*	2/30 (7)
*Primary cancer staging*	8/30 (27)
Concern for Thyroid carcinoma	3/30 (10)
Concern for Parotid carcinoma	2/30 (7)
Melanoma with cervical lymphadenopathy	2/30 (7)
Concern for soft palate carcinoma	1/30 (3)
*Follow-up imaging after cancer treatment*	15/30 (50)
Tonsil cancer	5/30 (17)
Thyroid cancer	3/30 (10)
Parotid cancer	3/30 (10)
Nasopharyngeal carcinoma	1/30 (3)
Oral tongue carcinoma	1/30 (3)
Plasmocytoma of the vocal cord	1/30 (3)
Sarcoma of the soft palate	1/30 (3)

Except where indicated, data represent the number of participants as a numerator/denominator with percentages in parentheses

* Data is presented as mean ± standard deviation

† Data represents a range of years

### Diffusion-weighted MR Protocol On the Study’s Scanners

Included patients underwent an institutional clinical protocol on either a 1.5 T scanner or a 3 T scanner. The institutional clinical protocol was not modified. Patients were scanned in the supine position using a 20-channel head and neck coil.

The DWI protocol included a low b‑value (b = 0 s/mm^2^ or 50 s/mm^2^) and a high b‑value (b = 800 s/mm^2^) with calculated ADC maps. After completion of the examination, images were reconstructed with the standard generalized autocalibrating partial parallel acquisitions-based processing (GRAPPA). Additionally, DL reconstruction of a reduced number of DWI averages was performed; one signal average for the low b‑value and three signal averages for the high b‑value for all scanners. Table 2 summarizes the acquisition parameters of the study scanners, the number of signal averages for DWI and DWI_DL_ and their acquisition time.

**Table 2:** Acquisition parameters of the study scanners for diffusion-weighted imaging (DWI), the number of averages for DWI and DWI_DL_ and their acquisition time

Acquisition parameters	1.5 T scanner*	3 T scanner **	3 T scanner ***
Repetition time	4500 ms	5100 ms	6100 ms
Echo time	59 ms	67 ms	83 ms
Slice thickness	4 mm	4 mm	4 mm
Fat saturation method	SPAIR	SPAIR	SPAIR
Acceleration factor	2 × 2 ^§^	2 ^§§^	2^§§^
Low b‑value	0 s/mm^2^	50 s/mm^2^	0 s/mm^2^
Signal averages for the low b‑value (DWI)	2 averages	2 averages	2 averages
Signal averages for the low b‑value (DWI_DL_)	1 average	1 average	1 average
High b‑value	800 s/mm^2^	800 s/mm^2^	800 s/mm^2^
Signal averages for the high b‑value (DWI)	12 averages	6 averages	8 averages
Signal averages for the high b‑value (DWI_DL_)	3 averages	3 averages	3 averages
Acquisition time for DWI	4 min 07 sec	2 min 15 sec	2 min 58 sec
Simulated Acquisition time for DWI_DL_	1 min 21 sec	1 min 13 sec	1 min 20 sec
Simulated reduction of acquisition time (%)	67.20%	45.93%	55.06%

*DWI* Diffusion-weighted imaging; *DWI*_*DL*_ Deep learning-reconstructed diffusion-weighted imaging; *SPAIR* SPectral Attenuated Inversion Recovery

§ with simultaneous multi-slice imaging (SMS) and generalized autocalibrating partial parallel acquisitions (GRAPPA).

§§ GRAPPA only

* MAGNETOM Sola, Siemens Healthineers

** MAGNETOM Vida, Siemens Healthineers

*** MAGNETOM Vida Fit, Siemens Healthineers

### Deep Learning-based DWI Reconstruction Algorithm

The DL DWI reconstruction algorithm utilized two DL-based reconstruction processes. First, raw complex frequency domain (k-space) to complex image domain DL reconstruction followed a variational network [16] where k‑space data, precalculated coil sensitivities and the sampling pattern served as input. A total of 17 unrolled iterations were performed. The first six iterations concentrated on the augmentation of missing k‑space data by maintaining data consistency without additional regularization. The remaining eleven iterations concentrated on denoising by including a network with hierarchical down-up architecture for regularization. The proprietary name of the DLR technique is “*Deep Resolve Boost*”. Second, image-based super resolution network *“Deep Resolve Sharp*” was employed to increase spatial resolution. The training of the raw complex frequency domain (k-space) to complex image domain DL reconstruction included 500,000 single-shot DWI images of different body regions acquired in volunteers at 1.5 T and 3 T clinical scanners (MAGNETOM scanners, Siemens Healthineers, Erlangen, Germany). The training pairs were generated by retrospectively doubling the acceleration factor i.e. reducing k‑space lines. The training of the image-based super resolution network was performed using a pixel shuffle network trained [17] on low-resolution images generated by down sampling the spatial resolution of the aforementioned dataset by factor two. All training was performed offline in PyTorch, the networks were then frozen and integrated in the reconstruction pipeline of the scanner in C++.

Finally, the DWI-specific reconstruction processes e.g. ADC calculation, were completed in an identical manner of standard DWI reconstruction.

**Fig. 2:**
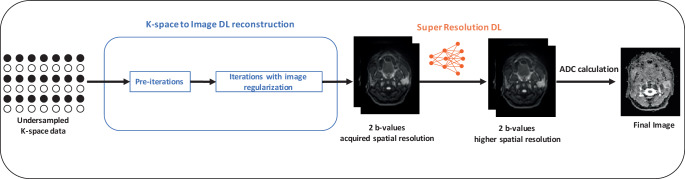
Schematic of the reconstruction pipeline of the deep learning DWI

Figure 2 summarizes the reconstruction pipeline of the utilized algorithm.

### Qualitative Image Readouts

Image readouts were performed by three readers. Two board-certified radiologists with eight years (S.A.) and five years (H.A.) of experience, and one radiology resident (J.M.B.) with two years of experience. DWI_S_ and DWI_DL_ with two b‑values and corresponding apparent diffusion coefficient (ADC) maps were available for study reading. The readers were blinded to the reconstruction type, clinical and radiological reports, and assessments made by their peers. Additionally, any patient- or sequence-specific identifiers were removed. To minimize recall bias, all readers evaluated the DWI and DWI-DL datasets in a randomized, mixed order during separate sessions, with a minimum washout period of two weeks between sessions. A dedicated workstation equipped with certified viewing software (Centricity PACS RA 1000; GE HealthCare, Chicago, Illinois, USA) was used under certified reading room conditions.

The outcome parameters for the image analysis were image sharpness, artifacts, noise, overall image quality, and diagnostic confidence.

The readers utilized a 4-point visual Likert score as follows:

Image sharpness (1: severely blurred; 2: moderately blurred; 3: slightly blurred; 4: no blurring). Image noise and artifacts were rated as follows: (1: severe artifacts and/or noise; 2: moderate degradation of image quality by artifacts and/or noise; 3: slight impediment of image quality by artifacts and/or noise; 4: no discernible artifacts/noise affecting image quality).

Overall image quality (1: non-diagnostic; 2: poor image quality; 3: good image quality; 4: excellent image quality). Diagnostic confidence (1: non-diagnostic; 2: poor diagnostic confidence; 3: good diagnostic confidence; 4: excellent diagnostic confidence).

### Quantitative Image Analysis

For the quantitative assessment of image quality, circular Region-of-Interests (ROI) with diameters of ≤ 10 mm were positioned bilaterally over the parotid glands and masseter muscles on the DWI images for each patient. Subsequently, these ROIs were automatically and computationally transferred to the corresponding DWI_DL_ images.

The quantitative outcome parameters were signal-to-noise ratio (SNR) and contrast-to-noise ratio (CNR), which were calculated using the following formulas, as described in previous studies [18–20].

SNR was defined as the ratio of the mean signal intensity of the parotid glands (SI _Parotid_) to the standard deviation of the signal intensity of the masseter muscles SD _Masseter_, which served as an indicator of the noise, Eq. 1.

1 $$\text{SNR\ }=\mathrm{SI}_{\mathrm{Parotid}}/\mathrm{SD}_{\mathrm{Masseter}}$$

SI _Parotid_ represents the averaged signal intensity of the parotid gland within the ROI placed on each side of the gland, and SD _Masseter_ denotes the average standard deviation of the signal intensity with the ROIs placed on each side of the master muscles.

CNR was calculated according to Eq. 2.

2 $$\mathrm{CNR}=\left(\mathrm{SI}_{\mathrm{Parotid}}-\mathrm{SI}_{\mathrm{Masseter}}\right)/\mathrm{SD}_{\mathrm{Masseter}}$$

Similarly, the ADC values from ROIs placed on the masseter muscles and parotid glands were extracted and compared.

Finally, ADC subtraction maps for two patients were computed using Image J software (1.54p, National Institute of Health, Wayne Rasband, USA).

### Statistical Analysis

Variables were reported as means, standard deviations (SD) and ranges and/or medians with interquartile ranges (IQR). Shapiro-Wilk test was used to assess data normality. The Wilcoxon signed-rank for paired data and paired t‑test were used to compare data from DWI and DWI_DL_. A *P* value < 0.05 was considered to indicate a statistically significant difference. Statistical analysis was performed using commercially available software (SPSS, IBM, version 28).

## Results

### Study Sample

A total of 30 patients with various pathologies of the head and neck region (mean age 55 ± 19 years (range 24–84), 18 men), with complete MRI datasets (DWI_S_ and DWI_DL_), were included. A total of 14 patients were scanned using a 1.5 T scanner (47%), and 16 patients were scanned using 3 T scanners (53%).

Patient demographics and clinical indications for the MRI examination are provided in Table 1. The top three indications were follow-up imaging after treatment (50%), primary cancer staging (27%), and evaluation of an indeterminate cervical mass (17%).

### Qualitative Image Readouts

Table 3 presents a comprehensive breakdown of the qualitative image analysis findings for both low b‑value and high b‑value images, along with ADC maps, as assessed by the three readers.

**Table 3:** Evaluation of image quality parameters in standard diffusion weighted imaging datasets (DWI) and deep learning reconstructed DWI datasets (DWI_DL_)

Outcome Parameters*	DWI^§^	DWI_DL_^§^	DWI^§§^	DWI_DL_^§§^	*P* value^†^
*Low b‑Value (B0/B50)* **					
Sharpness	3.22 ± 0.492 (2–4)	3.24 ± 0.504 (2–4)	3 (1)	3 (1)	0.157
Artifacts	3.27 ± 0.557 (2–4)	3.20 ± 0.584 (2–4)	3 (1)	3 (1)	0.109
Noise	3.27 ± 0.536 (2–4)	3.29 ± 0.525 (2–4)	3 (1)	3 (1)	0.414
Overall Image Quality	3.23 ± 0.520 (2–4)	3.26 ± 0.531 (2–4)	3 (1)	3 (1)	0.157
Diagnostic confidence	3.30 ± 0.550 (2–4)	3.28 ± 0.541 (2–4)	3 (1)	3 (1)	0.480
High b‑Value (B800) **					
Sharpness	3.26 ± 0.487 (2–4)	3.27 ± 0.493 (2–4)	3 (1)	3 (1)	0.655
Artifacts	3.23 ± 0.582 (2–4)	3.29 ± 0.604 (2–4)	3 (1)	3 (1)	0.275
Noise	3.24 ± 0.526 (2–4)	3.27 ± 0.515 (2–4)	3 (1)	3 (1)	0.157
Overall Image Quality	3.26 ± 0.552 (2–4)	3.32 ± 0.577 (2–4)	3 (1)	3 (1)	0.058
Diagnostic confidence	3.34 ± 0.523 (2–4)	3.32 ± 0.557 (2–4)	3 (1)	3 (1)	0.527
*ADC*					
Sharpness	3.04 ± 0.447 (2–4)	3.06 ± 0.459 (2–4)	3 (0)	3 (0)	0.564
Artifacts	3.01 ± 0.551 (2–4)	3.02 ± 0.653 (2–4)	3 (0)	3 (0)	0.847
Noise	2.97 ± 0.485 (2–4)	2.99 ± 0.462 (2–4)	3 (0)	3 (0)	0.157
Overall Image Quality	3.00 ± 0.497 (2–4)	3.03 ± 0.485 (2–4)	3 (0)	3 (0)	0.083
Diagnostic confidence	3.08 ± 0.479 (2–4)	3.12 ± 0.493 (2–4)	3 (0)	3 (0)	0.102

*DWI* diffusion-weighted imaging; *DWI*_*DL*_ deep learning-reconstructed diffusion-weighted imaging; *ADC* Apparent Diffusion Coefficient

* All tests are comparisons between the rating of the three readers as documented for DWI vs. DWI_DL_

** b‑values have the unit of s/mm^2^

§ Data for DWI and DWI_DL_ ratings are means ± standard deviation and ranges in parentheses.

§§ Data are medians, with IQRs in parentheses.

† Wilcoxon signed-rank test for ordinal variables.

**Fig. 3:**
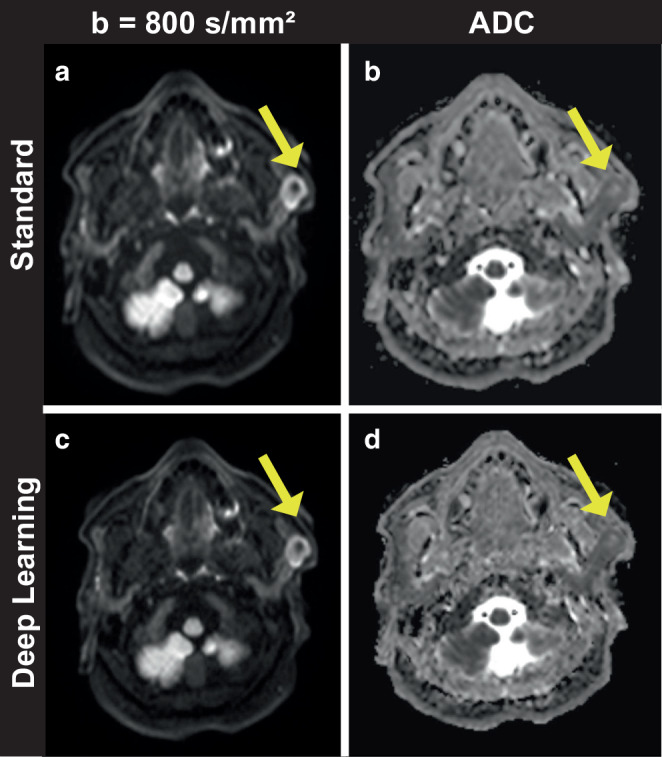
Diffusion-weighted imaging (DWI) at 1.5 T in a 67-year-old male with a histologically proven carcinoma of the left parotid gland. The first row (**a**, **b**) shows standard DWI (b = 800 s/mm^2^ and apparent diffusion coefficient (ADC)). The second row (**c**, **d**) shows the deep learning reconstructed images. The tumor is slightly hyperintense on the high b‑value DWI, corresponding to a low signal on the calculated ADC maps (yellow arrows). No significant differences between the images were noted despite the reduction of signal averages (from 12 averages to 3 averages, enabling an approximate reduction of acquisition time by 67%)

**Fig. 4:**
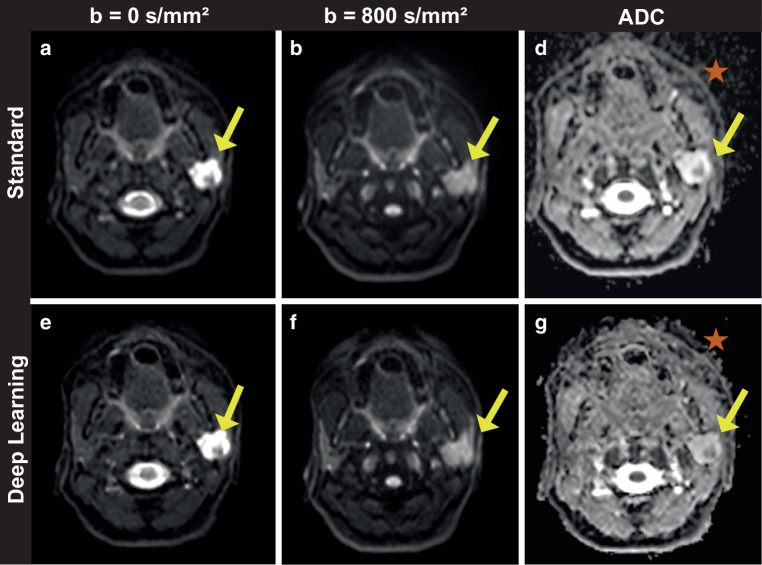
Diffusion-weighted imaging (DWI) at 1.5 T in a 43-year-old female with a histologically proven pleomorphic adenoma of the left parotid gland (yellow arrows). The first row (**a**, **b**, **d**) shows standard DWI with low and high b‑values (0 s/mm^2^, b = 800 s/mm^2^) and apparent diffusion coefficient (ADC). The second row (**e**, **f**, **g**) shows the deep-learning reconstructed images. The depiction of contours of the masticator muscles is slightly impaired in deep-learning reconstruction ADC; however, this does not affect diagnostic confidence (**f**). Otherwise, no significant differences between the images were noted

**Fig. 5:**
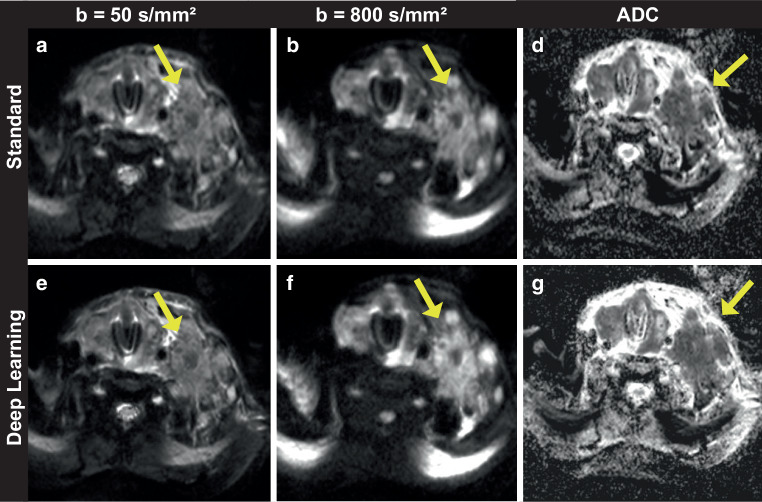
Diffusion-weighted imaging (DWI) at 3 T in an 84-year-old female with a histologically proven diffuse large B cell lymphoma of the left thyroid lobe (yellow arrows). The first row (**a**, **b**, **d**) shows standard DWI with low and high b‑values (50 s/mm^2^, b = 800 s/mm^2^) and apparent diffusion coefficient (ADC). The second row (**e**, **f**, **g**) shows the deep-learning reconstructed images. No significant differences between the images could be noted despite the reduction of signal averages

**Fig. 6:**
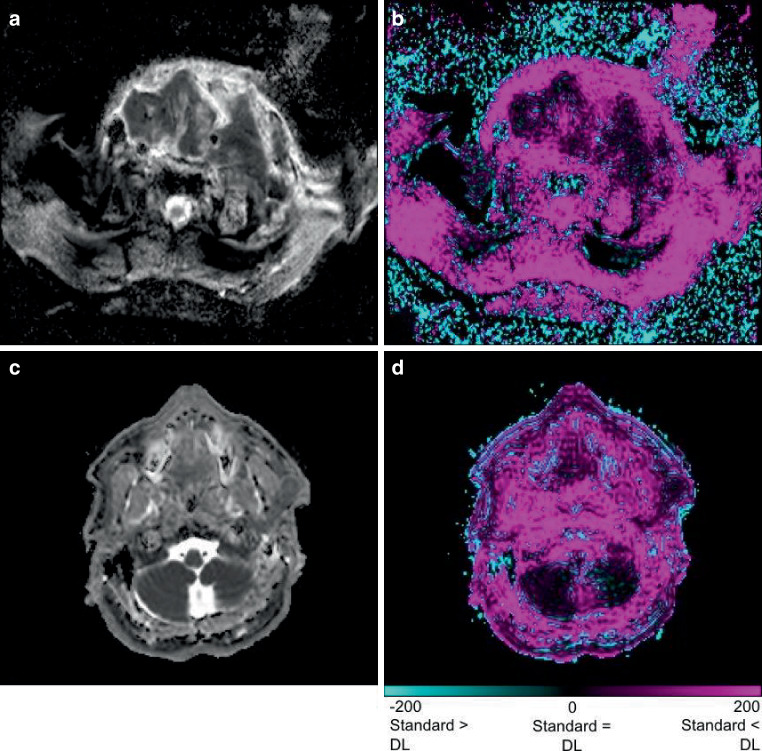
**a**, **c** Deep Learning-reconstructed Apparent Diffusion Coefficient (ADC) maps for two patients: (**a**) with diffuse large B cell lymphoma of the left thyroid lobe and (**b**) with parotid cancer. The corresponding ADC subtraction maps (DWI standard—DWI_DL_) are also shown. Notably, the noise distribution in the DL maps is much more homogeneous, particularly in panel (**b**), where the light blue noise in the subtraction map is evident to be much higher in the standard ADC, delineating the effect of denoising in DL images. Additionally, the pink color in the subtraction map represents areas of higher ADC values in DL map, as highlighted in the quantitative analysis

**Fig. 7:**
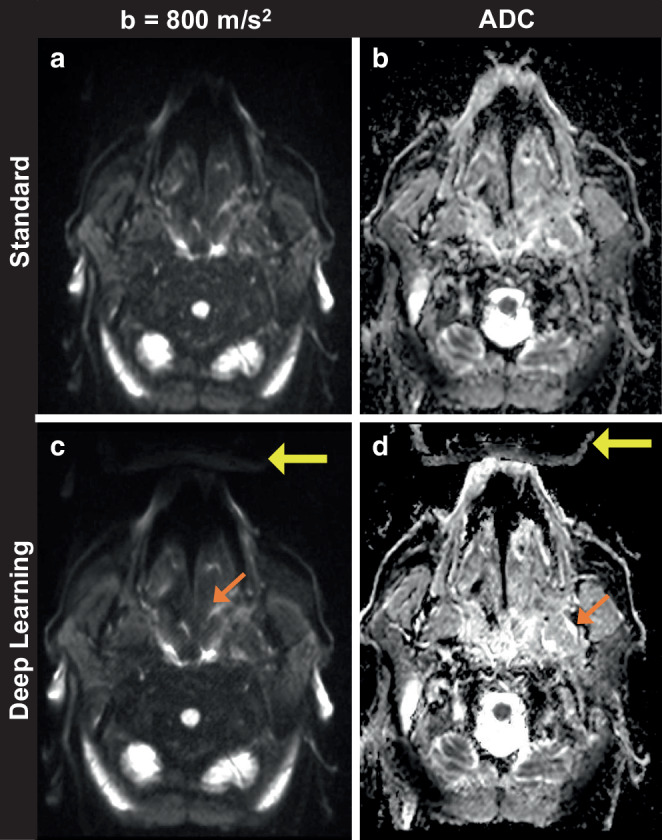
Diffusion-weighted imaging (DWI) at 3 T in a 69-year-old male undergoing a follow-up examination after radio- and chemotherapy due to tonsil cancer. The first row (**a**, **b**) shows standard DWI with a high b‑value (b = 800 s/mm^2^) and apparent diffusion coefficient (ADC). The second row (**c**, **d**) shows the deep learning reconstructed images. The deep learning reconstructed images show wrap-around artifacts (yellow arrows in **c**, **d**) and low-frequency diagonal parallel lines, probably due to the reduction of signal averages (brown arrows)

Figure 3 to 7 showcase examples of standard diffusion-weighted images (DWI) and deep-learning reconstructed DWI (DWI_DL_) from multiple patients exhibiting various pathologies.

Figure 3 displays images from a patient with histologically proven carcinoma of the parotid gland, while Fig. 4 depicts images from a patient diagnosed with histologically proven pleomorphic adenoma of the parotid gland. Fig. 5 presents images from a patient with histologically proven diffuse large B‑cell lymphoma of the left thyroid lobe.

Additionally, Fig. 6 represents ADC subtraction maps (ADC standard—ADC_DL_) of two patients. Finally, Fig. 7 exhibits images from a patient undergoing a follow-up examination after radio- and chemotherapy for tonsil cancer, revealing artifacts in the DL-reconstructed DWI (DWI_DL_).

In summary, across all readers, no evidence of statistically significant differences was observed between DWI and DWI_DL_ in terms of image sharpness, artifacts, noise, overall image quality, or diagnostic confidence, as shown in Table 3.

### Quantitative Image Analysis

The quantitative analysis revealed a minimal but statistically significant decrease in SNR and CNR in the deep learning-reconstructed images (*p* = 0.002 and *p* < 0.001, respectively) and a statistically significant increase in ADC values (*p* < 0.001) (Table 4).

**Table 4:** Comparison of Signal-to-Noise Ratio (SNR) and Contrast-to-Noise Ratio (CNR) as well as Apparent Diffusion Coefficient (ADC) values between DWI and DWI_DL_

Outcome Parameter	DWI^§^	DWI_DL_^§^	*P* value
SNR	14.7 ± 7.9 (3.1–29.7)	13.4 ± 7 (2.7–29.3)	*p* = 0.002^†^
CNR	6.7 ± 6 (−3.6–20.2)	5.7 ± 4.7 (−5.9–20)	*p* < 0.001^††^
ADC (Masseter muscle)	1366 ± 109 (1174–1738)	1501 ± 125 (1305–1830)	*p* < 0.001
ADC (Parotid gland)	969 ± 224 (203–1354)	1058.8 ± 218 (491–1434)	*p* < 0.001

*DWI* diffusion-weighted imaging; *DWI*_*DL*_ deep learning-reconstructed diffusion-weighted imaging

§ Data for the DWI and DWI_DL_ ratings are means ± standard deviation, and ranges in parentheses

† Wilcoxon signed-rank test for paired variables based on the Shapiro-Wilk test for normal distribution

†† Paired t‑test

## Discussion

This study investigated the clinical feasibility of a novel deep-learning reconstruction of diffusion-weighted MR imaging (DWI_DL_) of the head and neck at 1.5 T and 3 T. Our findings show that DWI_DL_ enabled an approximate 67% reduction in examination time at 1.5 T and up to 55% at 3 T without compromising image quality or diagnostic confidence. Despite the relevant reduction of signal averages, image noise and artifacts were not significantly impacted.

The added value of an MRI examination has multiple definitions [21]. A fast and efficient MRI examination with diagnostic quality offers significant added value by improving patient experience and optimizing scanner throughput [22]. Especially in head and neck DWI, high-quality examinations are essential for detecting subtle findings within a region of complex anatomy. This is particularly challenging due to limitations such as reduced spatial resolution, magnetic field inhomogeneities, and motion artifacts. These issues are even more pronounced in patients who are often elderly, have multiple comorbidities, and may be restless during longer scan times. Therefore, achieving comparable image quality with reduced examination time is highly beneficial. While DWI is generally robust against motion artifacts, minimizing scan duration can further improve detection reliability in this vulnerable patient population.

Recently, although still sparse, emerging evidence has supported the implementation of deep-learning reconstruction techniques in abdominal, prostate and neuroimaging [3, 9, 13–15].

Our findings partly corroborate the results of these few published studies [3, 9, 13–15]. For instance, Afat et al. utilized a DWI_DL_ algorithm in the context of liver imaging at 1.5 T [23]. The authors retrospectively reduced signal averages from 12 averages (high b‑value) to 6 averages, resulting in approximately a 42% reduction in acquisition time. Similar to our study, the authors found no significant differences between DWI_DL_ and standard DWI in terms of artifacts and overall image quality or diagnostic confidence for both b‑values as well as ADC maps.

In prostate imaging, Ueda et al. [14] retrospectively applied the DL-reconstruction algorithm for prostate DWI-MRI at 3 T and showed that it improved the image quality. However, this was done without accelerating the standard DWI. Similarly, Ursprung et al. retrospectively deployed a DL-reconstruction algorithm for prostate DWI-MRI at 3 T, but they accelerated the original examination by approximately 40%. Despite the reduction of signal averages, the original image quality was maintained, and less image noise was noted in the DWI_DL_ images. Similar results were found by Johnson et al., where four readers found no differences in image quality between accelerated DL-reconstructed images and standard DWI-MR of the prostate. [24].

In neuroimaging, Altmann et al. [15] conducted a prospective study of 85 patients and implemented DL reconstruction of DWI and concluded that highly accelerated brain DWI was possible while increasing image quality.

Finally, a retrospective study by Fujima et al. [8] analyzed the impact of a model-based DL reconstruction algorithm in head and neck DWI. The authors noted that DWI_DL_ enabled a significant improvement of image quality and denoising. However, the authors did not accelerate the original exam.

In our study, we found that image quality and diagnostic confidence did not improve, but neither did they deteriorate. This outcome may be attributed to different scanning protocols, different parallel imaging factors and techniques, and diverse hardware to scan different organs. Most importantly, these differences could be due to the aggressive reduction of signal averages employed in our study, especially at 1.5 T. This approach allowed for greater scan time reduction compared to most published studies, yet image quality and diagnostic confidence remained comparable to the fully sampled standard DWI.

It is important to note that, in some cases, wrap-around artifacts and low-frequency stripe artifacts were observed in the deep learning (DL)-reconstructed images (Fig. 6), likely related to signal loss resulting from the reduced number of signal averages. While the exact cause requires further investigation in future studies, these artifacts were readily identifiable by the readers in our cohort and did not significantly impact diagnostic confidence. Given that this study represents a first feasibility assessment in the diagnostically challenging field of head and neck MRI, further research is warranted to better understand and address these artifact patterns.

Finally, the quantitative analysis revealed a minimal but statistically significant decrease in SNR and CNR in the deep learning-reconstructed images (Table 4). These results should be interpreted with caution for two main reasons. First, due to the slight difference observed and the small patient sample size, this difference might not have reached statistical significance in a larger cohort. Second, ROI measurements performed on retrospectively generated images without phantom validation may lack reliability; it has been suggested that using a non-linear reconstruction technique might lead to unreliable ROI-based measurements [25, 26]. This finding could also be explained by the significant reduction in signal averages, which led to a notable drop in signal intensity. However, this loss was compensated for by the deep learning reconstruction, as the qualitative analysis showed no differences between the conventional DWI and the deep learning-reconstructed images.

Also, our ROI-based analysis revealed that ADC values were higher in DWI_DL_ than in DWI. Deep learning-reconstructed DWI can yield different ADC values compared to conventional DWI, with previous studies reporting higher [27], similar [28], or lower measurements [29] depending on the anatomical region, imaging protocol, and reconstruction algorithm used.

It has been suggested that ADC values in DWI_DL_ may be higher than those in standard DWI due to improved noise reduction and protocol differences that influence signal characteristics. However, the variability in ADC values is not yet fully understood and may be influenced by differences in acquisition protocols, anatomical regions, and artifacts introduced during imaging (e.g., motion artifacts in liver imaging versus geometric distortions in head and neck imaging) [29]. Future studies are warranted to further elucidate this phenomenon.

This study has limitations. The retrospective, single-center design introduces an inherent selection bias in a study with a small sample size. Also, retrospective DL reconstruction does not truly account for motion artifacts that might be reduced due to the actual prospective acceleration of undersampled MR examinations [30]. Furthermore, DLR reconstructions were generated directly from the accelerated acquisitions. Consequently, a separate evaluation of undersampled reconstructions without DLR was not feasible with the current reconstruction pipeline. However, one advantage of the retrospective design is that a reproducible intraindividual comparison could be achieved due to the fact that the original dataset is identical and the data is intrinsically co-registered [3]. Furthermore, the included MRI scanners and DL reconstruction algorithm were from a single vendor. However, including DL reconstructions on 1.5 T and 3 T might help, at least partially, to increase the generalizability of our findings. Finally, our protocol relied on increasing the number of averages to improve SNR, whereas techniques such as multi-shot EPI (REadout Segmentation Of Long Variable Echo trains e.g., RESOLVE) could more effectively mitigate susceptibility and motion-related artifacts. We also note that RESOLVE was not available on our scanners at the time of the study, but represents a promising direction for future protocol optimization.

Nonetheless, our preliminary findings show that the implementation of the deep-learning reconstruction of retrospectively accelerated DWI in the context of head and neck imaging is feasible and paves the way for future clinical studies and potential clinical applications. A future direction would be to conduct a prospective study with a larger sample size and with prospective deployment of the DL algorithm on undersampled data, where the clinical impact of this technique can be further explored. An interesting future direction is also to explore the impact of DL reconstruction on readout multi-shot segmented k‑space acquisition sequences, which deliver better image quality and less distortion.

In conclusion, deep learning reconstruction of diffusion-weighted MRI of the head and neck is feasible, enabling an approximate 67% reduction in examination time at 1.5 T and up to 55% at 3 T without compromising image quality or diagnostic confidence. This technique sets the stage for a significantly accelerated MRI of the head and neck with diagnostic quality.
